# The Balloon-Pushing Technique to Rescue a Covered Hypogastric Artery During Endovascular Aortic Repair

**DOI:** 10.1177/15266028251384923

**Published:** 2025-10-22

**Authors:** Jose I. Torrealba, Giuseppe Panuccio, Petroula Nana, Daour Yousef Al Sarhan, Nils Zabel, Tilo Kölbel

**Affiliations:** 1 German Aortic Center Hamburg, Department of Vascular Medicine, University Heart Center, University Hospital Hamburg Eppendorf, Hamburg, Germany

**Keywords:** EVAR, hypogastric artery, coverage hypogastric artery, iliac limbs, bailout

## Abstract

**Introduction:**

The coverage of a hypogastric artery (HA) may lead to several complications, such as gluteal claudication, erectile dysfunction, and even a higher risk of spinal cord ischemia in complex aortic repair. It has been shown to occur in 1% to 8% of endovascular aortic repair. We present the balloon-pushing technique to rescue an HA after its overstenting by an iliac limb.

**Technique:**

After the HA overstenting, a 20 Fr sheath was kept close to the distal edge of the iliac limb. A stiff wire was kept in place and a 12×40 mm balloon was inflated at the overlapping between the iliac limb and the main endograft. Then, the balloon shaft was vigorously pushed, creating a posterior and superior movement of the iliac limb, that ended up freeing the HA origin.

**Conclusion:**

The balloon-pushing technique is a simple technique to rescue the HA after its coverage by an iliac limb. Patient anatomy and knowledge of the endografts must be carefully considered.

**Clinical Impact:**

The balloon-pushing technique is a simple technique to rescue the hypogastric artery after its coverage by an iliac limb. Despite its simplicity the technique has not been previously described. We believe it should be used as first manouver to rescue the hypogastric artery if covered by 5-10 mm.

## Introduction

The unintended coverage of a hypogastric artery (HA) during iliac limb deployment may lead to several complications after endovascular aortic repair (EVAR), such as buttock claudication or erectile dysfunction in up to 50% of patients.^1^ Preserving the HA becomes particularly important in complex aortic repairs using fenestrated or branched endovascular techniques, as these procedures involve the coverage of longer segments of the aorta, with an increased risk of spinal cord ischemia if collateral pathways—including the hypogastric circulation—are not patent.^2^

Therefore, in case of an accidental HA coverage, it is desirable to regain unimpeded perfusion to this arterial territory. However, despite their clinical significance, only anecdotal evidence exists regarding available techniques to rescue the HA.^3^

We present a technical note on the use of the “balloon-pushing technique” to rescue an inadvertently occluded HA.

## Technical Note

Consent for the publication of the case was granted by the patient.

**Figure 1.**
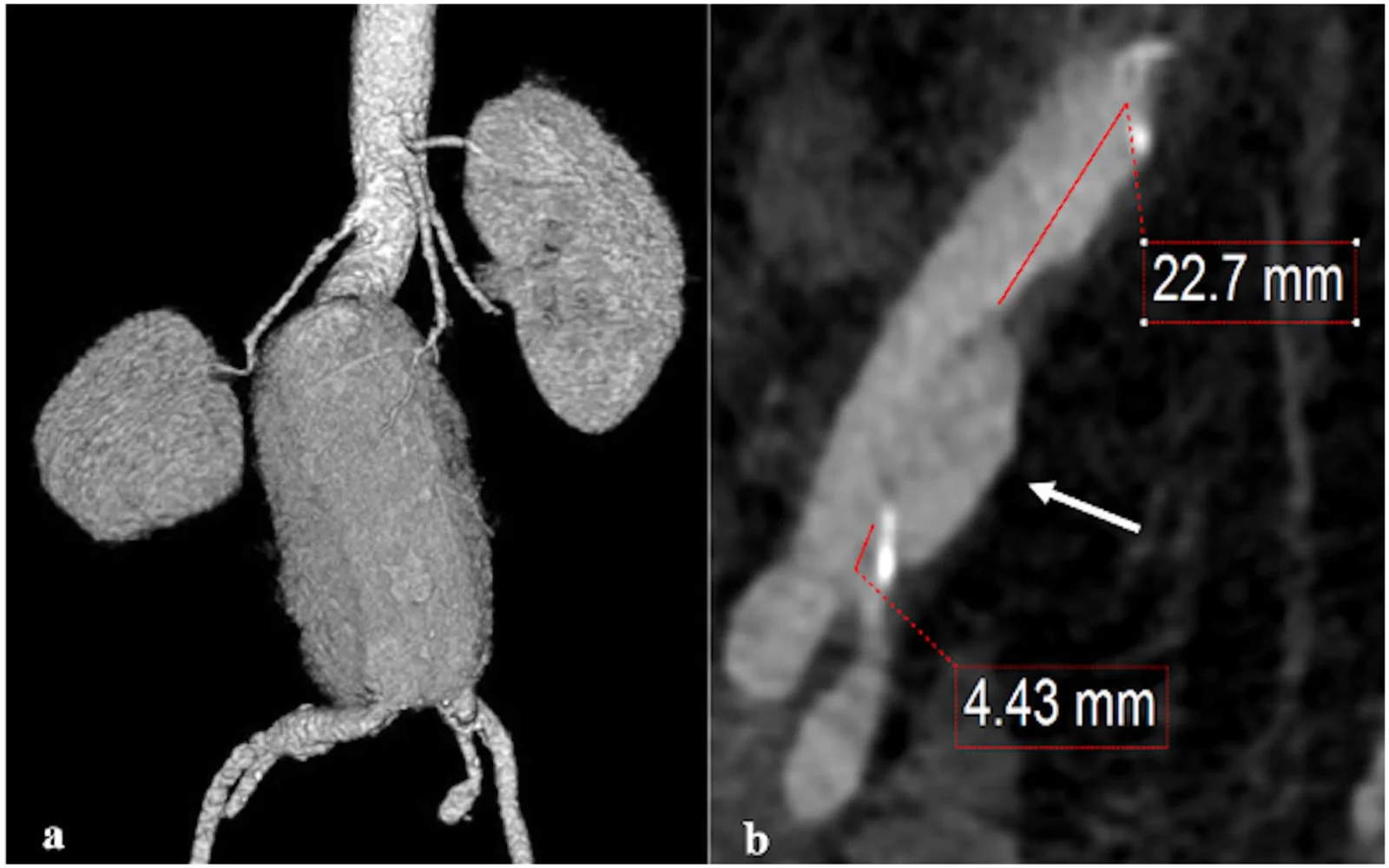
(a) 3D reconstruction of the AAA, (b) Right CIA with penetrating ulcer (white arrow) and proximal and distal landing zone lengths.

A 79-year-old patient with a history of chronic obstructive pulmonary disease (COPD) and a known abdominal aortic aneurysm (AAA) diagnosed 20 years ago presented at follow-up with a computed tomography angiography (CTA) showing a 6.5 cm infrarenal aortic aneurysm. The aneurysm had a 23 mm long proximal neck; however, the distal right common iliac artery (CIA) showed an 18 mm penetrating ulcer, with a 2 cm proximal seal zone and a limited distal landing zone of about 4 mm (Figure 1). Endovascular repair with a standard infrarenal endograft was planned. Based on its diameter, the dilated right CIA did not itself meet criteria for repair. Nevertheless, our strategy was to land the iliac limb as close as possible to the iliac bifurcation, aiming to exclude the penetrating ulcer without compromising the sealing zone, which was anticipated to be achieved proximal to the dilated segment of the CIA.

## Procedure

Under general anesthesia, bilateral percutaneous femoral access was obtained. Through the right access, a Lunderquist wire (Cook Medical, Bjaeverskov, Denmark) was advanced to the thoracic aorta, and a 28 mm proximal Zenith bifurcated endograft (TFFB 28-111, Cook Medical Bjaeverskov, Denmark) was deployed just below the renal arteries. Then, a 16 mm ZSLE Zenith limb (Cook Medical) was deployed in the left CIA above the iliac bifurcation. On the right side, after confirming the distance to the iliac bifurcation, a 13- to 90-mm ZSLE iliac limb (Cook Medical) was deployed as close as possible to the bifurcation in order to cover the penetrating ulcer in the CIA. A control angiography revealed that the right limb had been deployed into the external iliac artery (EIA), covering the origin of the HA.

**Figure 2.**
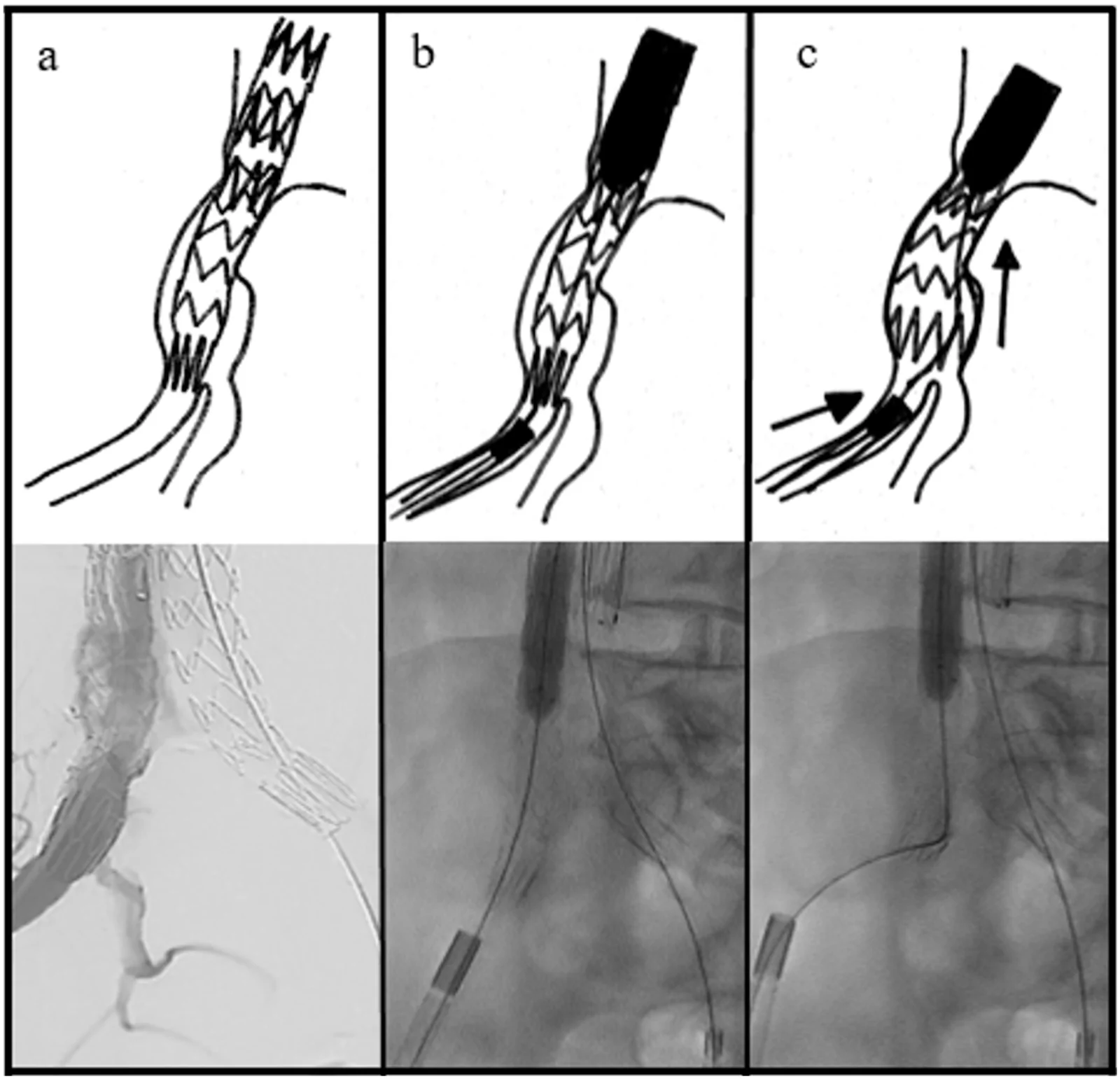
Schematic drawing and fluoroscopic images of the Balloon-Pushing Technique. (a) Origin of the HA covered by the iliac limb; (b) Inflation of the anchoring balloon at the overlap between main endograft and iliac limb, the big profile sheath is approached to the distal end of the iliac limb; and (c) vigorous push of the balloon shaft with superior and posterior movement of the iliac limb.

**Figure 3.**
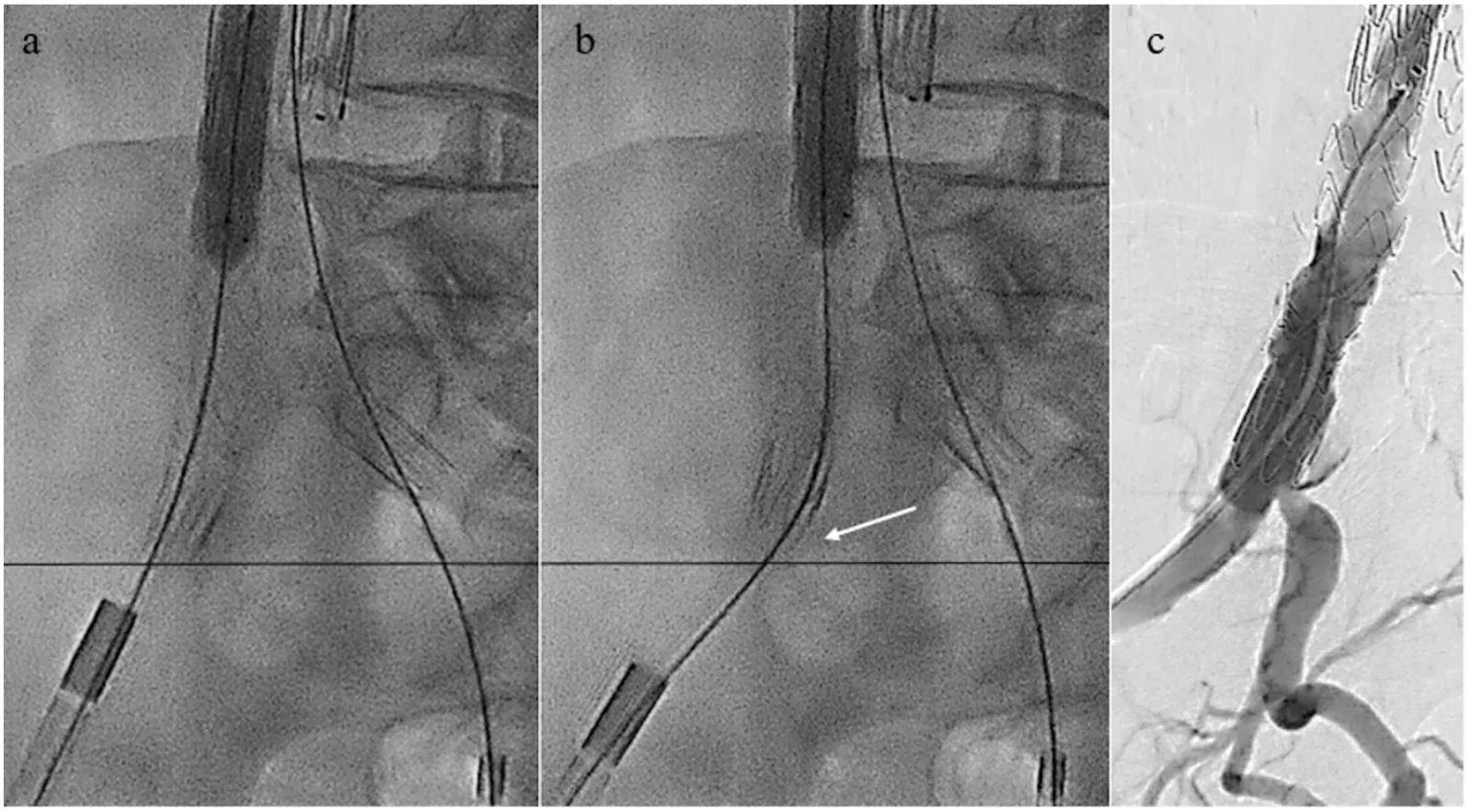
(a, b) same projection fluoroscopy before (a) and after (b) the Balloon-Pushing Technique. White arrow signals proximal displacement of the graft by several millimeters after the Balloon-Pushing Technique; and (c) DSA showing a free HA origin.

**Figure 4.**
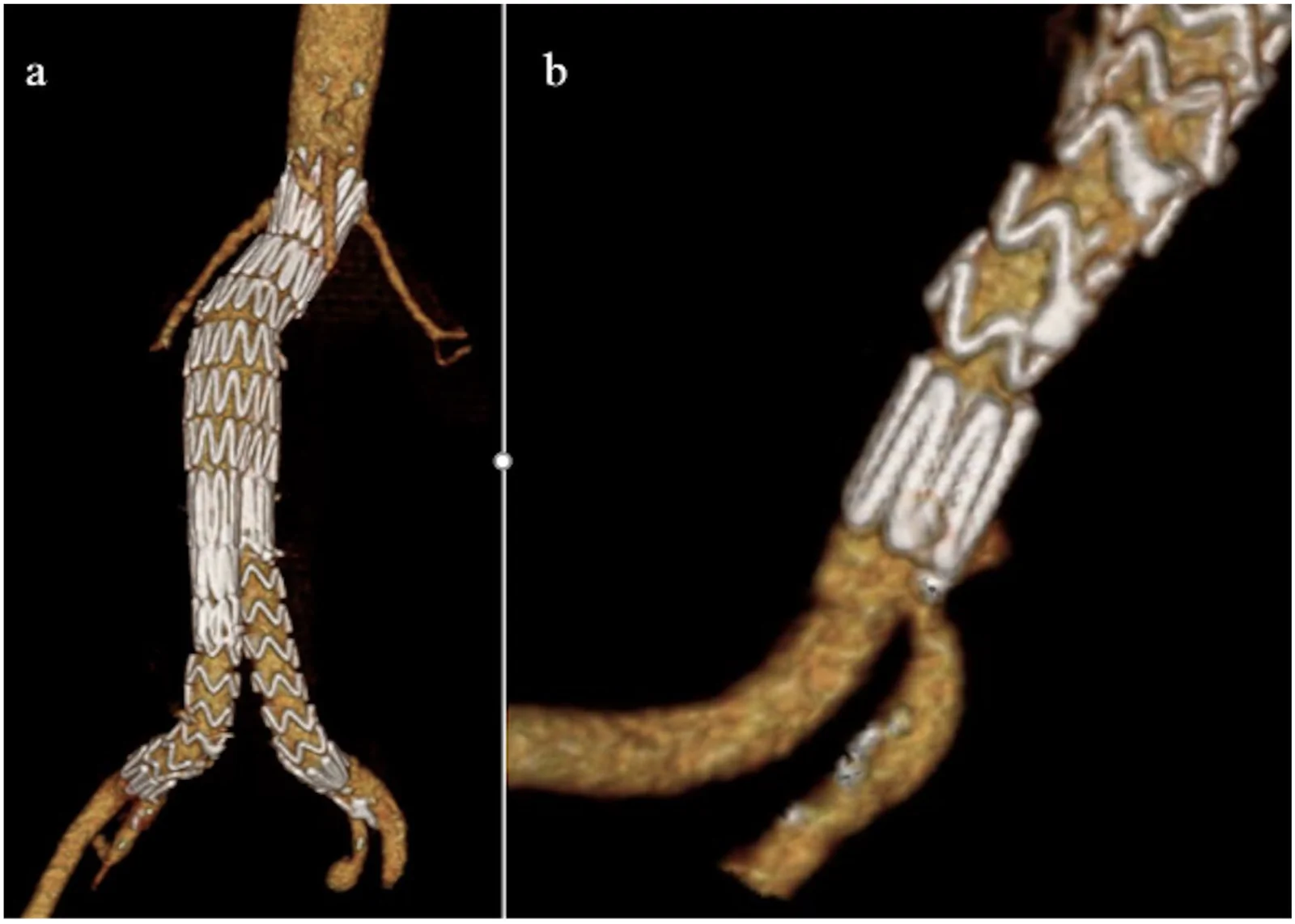
3D reconstruction. (a) EVAR control (b) Left HA with unobstructed perfusion.

As a bailout maneuver, the balloon-pushing technique was used to restore the patency of the right HA. The 20 Fr sheath from the main endograft delivery system was advanced in proximity to the distal end of the right iliac limb. Keeping the stiff Lunderquist wire in place, a 12×40 mm balloon was introduced and fully inflated at the overlapping zone between the main bifurcated endograft and the iliac limb. While maintaining constant forward pressure on the sheath, the balloon shaft was vigorously pushed, creating a posterior and upward movement of the iliac limb (Figure 2). After this maneuver, a control angiography showed a now-free ostium of the HA, with fluoroscopic imaging revealing several millimeters of proximal displacement of the iliac limb (Figure 3). The postoperative course was uneventful, and a pre-discharge CTA showed adequate sealing of the graft with an unobstructed HA ostium (Figure 4).

## Discussion

Overstenting of the HA origin may be related to dynamic deformations of the iliac artery caused by the presence of stiff devices, such as wires, sheaths, and endograft delivery systems, as well as to inaccuracies in iliac length sizing or endograft deployment. In our case, we believe that the attempt to achieve precise coverage up to the iliac bifurcation prompted a high-risk deployment of the iliac limb, ultimately resulting in inadvertent coverage of the HA origin. Based on large EVAR series, the unintended coverage of the HA has been shown to occur in 1% to 8% of cases.^4–6^ However, detailed analysis of causes or consequences has been directly addressed only by a few publications.^3,4,7–9^ Moreover, evidence regarding the management of this complication remains limited. The simplest approach may be conservative treatment without revascularization of the HA, which—aside from the risks of gluteal claudication and erectile dysfunction—is often well tolerated.^10^ However, in a minority of cases, particularly in patients with higher-risk features, such as contralateral HA occlusion or extensive aortic repair, HA occlusion can lead to serious ischemic complications, including colonic, gluteal, or spinal cord ischemia.^10^ Given that there is still no clear consensus on which patients are most vulnerable to adverse outcomes following HA occlusion—and considering that we frequently treat patients undergoing extensive EVAR with fenestrated or branched EVAR—it is our practice to attempt preservation of all HAs whenever possible.

To date, only a single case report has been identified regarding interventional treatment of unintended HA coverage. Wheatley^3^ reported its revascularization via an in-situ fenestration of an iliac limb covering the origin of the HA. Although the result was successful, this technique demands high endovascular skills, especially in narrow or tortuous iliac arteries, where the correct position of the catheter or puncturing needle might make this procedure very challenging.

As demonstrated by the use of parallel stenting to rescue covered renal arteries,^11,12^ a similar approach can be applied to the HA. Hsu et al^13^ reported catheterization of a covered HA through a paragraft approach, entering retrogradely between the graft and the vessel wall and eventually coiling the HA. However, given that the EIA measures in average of 9 to 11 mm in diameter,^14,15^ the implantation of an HA stent parallel to the iliac limb at this level could cause space constraints, potentially leading to thrombosis or complicating future arterial access.

In contrast, the balloon-pushing technique is a relatively straightforward maneuver. It relies on a static fixation point created by an inflated non-compliant balloon between the main graft and the iliac limb, which prevents cranial migration of the limb. With a stiff wire in place, a longitudinal force is applied along the graft’s axis, which bends the distal portion of the limb. This translates the graft posteriorly and proximally, allowing it to conform to the vessel curvature and freeing the HA ostium (Fig. 2).

Interacting physical forces—such as torque along the curved vessel, longitudinal force, and the graft’s elasticity and plasticity—allow cranial movement of the limb by approximately 5 to 10 mm, based on our experience.

The balloon-pushing technique is not without risks. With sufficient radial force from the proximal endograft against the aortic neck, and with the contralateral limb already deployed and secured, there should be adequate stability to advance only the iliac limb upward. However, in cases where the proximal graft diameter is inadequately oversized, forceful advancement of the sheath may result in displacement of the entire graft upward. Another potential complication is also the cranial displacement of the entire iliac limb, a risk that may be increased by the use of compliant or undersized balloons, which offer less secure fixation. It is particularly important to avoid cranial displacement of an iliac limb during fenestrated endovascular aortic repair (FEVAR), especially when renal artery stents are positioned near the aortic bifurcation. This might be the case with the use of bifurcated grafts with inverted limbs or when the bifurcation is incorporated into the FEVAR graft, where upward displacement of an iliac limb can damage the adjacent renal stents. Consequently, it is essential to be familiar with the specific characteristics of the endografts being used, as differences in the diameters of iliac limbs—both in main body endografts and in iliac extensions—across manufacturers may require the selection of different balloon sizes to achieve optimal iliac limb fixation.

There is also a risk of EIA damage caused by the high-profile sheath, which could result in dissection or rupture, particularly in small-diameter EIAs or in cases with acute EIA take-off angles. Additional risks include intimal damage or even HA thrombosis due to dragging the iliac limb upward—especially in the presence of severe atherosclerosis or calcification of the CIA or HA origin.

In cases of inadvertent HA coverage by the iliac limb—as long as the coverage is limited to a few millimeters—the balloon-pushing technique offers a simple and rapid solution. Nonetheless, individual patient anatomy and the above-mentioned risk factors must be carefully considered when planning the intervention. Further research might help to clarify the aforementioned potential risks and their contributing factors.
